# Clonal Dissemination of Multidrug-Resistant and Hypervirulent *Klebsiella pneumoniae* Clonal Complex in a Chinese Hospital

**DOI:** 10.3390/pathogens11101202

**Published:** 2022-10-18

**Authors:** Yi Wang, Mingxi Hua, Jingqiao Wang, Wen Xing, Jiatian Chen, Jingyuan Liu, Pengcheng Du

**Affiliations:** 1 Department of Clinical Laboratory, Beijing Bo’ai Hospital, China Rehabili-Tation Research Center, Capital Medical University, Beijing 100068, China; 2 Biomedical Innovation Center, Beijing Shijitan Hospital, Capital Medical University, Beijing 100038, China; 3 Department of Critical Care Medicine, Beijing Ditan Hospital, Capital Medical University, Beijing 100015, China; 4 Institute of Infectious Diseases, Beijing Ditan Hospital, Capital Medical University, Beijing 100015, China

**Keywords:** *Klebsiella pneumoniae*, clonal complex 15, hypervirulence, antimicrobial resistance, whole genome sequencing

## Abstract

The emergence of high antimicrobial-resistant and hypervirulent *Klebsiella pneumoniae* (hvKp) clones in clinics has become a cause of concern in recent years. Despite the global spread of the clonal complex (CC) 258, hvKp of other non-CC258 subgroups also emerged. Here, by performing a retrospective study from July 2019 to August 2020 in a Chinese hospital, we obtained 25 *K. pneumoniae* isolates belonging to CC15. By antimicrobial susceptibility testing and whole genome sequencing and analysis, we obtained the resistant phenotypes and genotypes of these isolates. Twenty-one isolates (84%) were carbapenem-resistant, and eighteen were *bla*_KPC-2_ positive. In addition, ten isolates were identified as putative hvKp and seven were carbapenem-resistant hvKp. Nine isolates carried the pLVPK-like virulence plasmid, which contains the fragment including *rmpA2*, *peg-589*, *iutA*, and *iucABCD*. Another isolate carried *iucA*. Phylogenetic analysis revealed that the isolates belonged to four lineages, and the putative hvKp isolates were identified in three of these. Two independent sublineages of putative hvKp were caused by the acquisition of pLVPK-like virulence plasmid. Based on comparative genomic analysis, the number of pairwise single nucleotide polymorphisms amongst the four sublineages, Lineage 1a, 1b, 2a, and 2b, were 1–43, 2–13, 129–279, and 3–4, respectively, indicating clonal transmission of Lineage 1a, 1b, and 2b. These results indicate that multiple lineages of CC15 carbapenem-resistant hvKp have emerged in the hospital and caused nosocomial transmission, and that the spreading of virulence plasmids among classic *K. pneumoniae* subtypes might become more common and happen more easily. These findings highlight the importance of surveillance of local epidemics of non-CC258 subgroups in hospitals.

## 1. Introduction

*Klebsiella pneumoniae*, a clinically important Gram-negative bacterium [1], is one of the bacteria in the infamous ESKAPE group of superbugs most prevalent in healthcare settings and that causes a large number of healthcare-associated infection cases worldwide [2]. In addition to the increasing prevalence of multidrug-resistant (MDR) strains (e.g., the clonal complex [CC] 258 including multilocus sequence type [ST] ST258, ST11, ST340, ST437, and ST512), the emergence of more and more hypervirulent *K. pneumoniae* (hvKp) clones in clinics has been cause of concern [3,4]. Previously, hvKp strains had been mostly isolated from the community-acquired infections cases, like liver abscess patients, and most were antimicrobial sensitive. However, the epidemiology of hvKp has been changing in recent years, together with its resistance phenotype [5].

In 2017, Gu et al. first reported the emergence of a ST11 hvKp clone presenting both hypervirulence and carbapenem resistance, which caused a fatal outbreak in a Chinese hospital [6]. Since then, this carbapenem-resistant hvKp (cr-hvKp) has spread worldwide. In addition, more and more hvKp subtypes have been reported in hospitals, including ST258, ST11, ST15, and ST23, and have commonly acquired high antimicrobial resistance [7]. In various regions of China, these subtypes have also become prevalent in hospitals [8]. These clinically prevalent hvKp commonly present high rates of antimicrobial resistance, which makes the clinical situation more complicated. In addition, a group of non-CC258 subtypes has been continually spreading in hospitals [9]. CC15, mainly consisting of ST14 and ST15, has become one of the most prevalent among the hvKp subtypes [10]. Among these subgroups, multiple drug resistance (MDR), and even extensive drug resistance, has become more and more common worldwide [3,4].

Therefore, the increasing prevalence of hvKp clones calls for continuous surveillance of this superbug in healthcare settings. In this study, we performed surveillance of nosocomial infections caused by *K. pneumoniae* and found that CC15 was the second prevalent subtype in our hospital. The genome sequences were obtained by whole genome sequencing (WGS), and the potential nosocomial transmission of the isolates and their genomic characteristics were explored. Notably, we found that multiple lineages of CC15 cr-hvKp had emerged in our hospital, suggesting that the spreading of virulence plasmids among classic *K. pneumoniae* (cKp) subtypes might become more common and happen more easily. These results highlight the importance of surveillance for the CC15 subtype.

## 2. Materials and Methods

### 2.1. Strain Collection

We performed a retrospective study from July 2019 to August 2020 in Beijing Bo’ai Hospital, a Class A tertiary hospital in Beijing, China. Strains were cultured on Columbia blood agar plate. The monoclonal strains were selected for identification by the Vitek compact 2 system. All of the *K. pneumoniae* strains were stored at −80 °C. Nosocomial infection cases were identified as previously described [5].

### 2.2. Antimicrobial Susceptibility Testing

The antimicrobial susceptibility testing was performed according to the 2020 Clinical and Laboratory Standards Institute (CLSI) guidelines, using the following agents: Amikacin (AMK), Ciprofloxacin (CIP), Imipenem (IPM), Levofloxacin (LEV), Tobramycin (TOB), Ampicillin (AMP), Ampicillin/Sulbactam (SAM), Aztreonam (ATM), Cefepime (FEP), Cefotetan (CTT), Ceftazidime (CAZ), Ceftriaxone (CRO), Piperacillin/Tazobactam (TZP), and Trimethoprim/Sulfamethoxazole (SXT). The results were interpreted following the Clinical and Laboratory Standards Institute (CLSI) guidelines. The strain presenting IPM resistance was defined as crKp. The MDR phenotype was defined as being resistant to three or more different classes of antimicrobial agents [5].

### 2.3. Whole Genome Sequencing, De Novo Assembly, and Annotation

The genomic DNA of all isolates was extracted with the TIANamp Bacteria DNA Kit (Cat. no. DP302, Tiangen, China), and then sequenced using the NovaSeq 6000 platform (Illumina, San Diego, CA, USA) by constructing paired-end libraries with an average insert size of 500 bp to obtain 150 bp reads. We used the fastQC software to obtain the clean data from the raw data and assembled the clean data with SPAdes v3.15.2 [11,12]. The draft genome sequences were subsequently annotated using Prokka [13].

The multi-locus sequence types and capsular types of the isolates were analyzed using Kelaborate [14]. Antimicrobial resistance genes, virulence genes, and plasmid replicon types were annotated by comparison with relevant databases, including ResFinder [15], Virulence Factor Database [16], ISfinder [17], and plasmidFinder [18], using the BLAST software [19]. We selected thresholds of 90% identity and minimum length coverage of 80% to identify antimicrobial resistance and virulence genes. The strains carrying at least one of the genes *peg-344*, *iroB*, *iucA*, *rmpA*, and *rmpA2* were defined as putative hvKp, while others as cKp.

### 2.4. Phylogenetic Analysis

As part of our phylogenetic analysis, the sequencing reads were mapped to the reference sequence of *K. pneumoniae* strain BR (ST15, accession no.: CP015990) using Bowtie 2 v2.2.8 (https://github.com/BenLangmead/bowtie2, accessed on 10 October 2022), and the single nucleotide polymorphisms (SNPs) were analyzed with Samtools v1.9 and combined using the iSNV-calling pipeline (https://github.com/generality/iSNV-calling, accessed on 10 October, 2022) we previously constructed. High-quality SNPs supported by more than five reads of mapping quality >20 were retained. The recombination sites were subsequently detected by Gubbins [20]. The concatenated sequences of filtered polymorphic sites conserved in all the Kp strains (core genome SNPs, cgSNPs) were used to perform phylogenetic analysis using the maximum likelihood method by IQ-Tree [21].

### 2.5. Data Availability

All sequencing data have been deposited in the NCBI GenBank database under accession number PRJNA881845.

## 3. Results

### 3.1. Sources of CC15 K. pneumoniae Isolates and Their Antimicrobial Susceptibility Profiles

**Figure 1:**
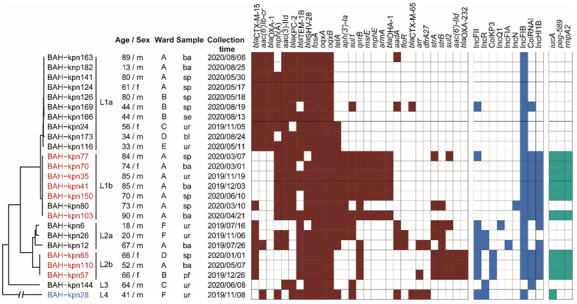
Phylogenetic analysis of *K. pneumoniae* CC15 isolates in our hospital correlated with the distribution of resistance genes (red–white heatmap), virulence genes (blue–white heatmap) and plasmid types (green–white heatmap). Six wards were included (A: department of respiratory medicine; B: intensive care unit; C: department of neurological rehabilitation I; D: department of neurosurgery; E: department of neurological rehabilitation II; F: department of spinal and neural functional reconstruction). The isolates are from six types of samples (ba: bronchoalveolar lavage fluid; bl: blood; pf: pleural fluid; se: secretion; sp: sputum; ur: urine). The putative cr-hvKp isolates are in red and the one putative hvKp isolate is in blue.

We identified 25 non-repetitive CC15 Kp isolates during the one-year monitoring of our hospital (Figure 1). CC15 was the second prevalent subgroup after CC258. The isolates were obtained from 22 patients hospitalized in six wards, of which there were 18 males and 4 females, with a median age of 66 (IQR: 44–80). The samples from which the isolates were cultured were sputum (8 isolates), bronchoalveolar lavage fluid (7 isolates), urine (7 isolates), blood (1 isolate), pleural fluid (1 isolate) and secretion (1 isolate).

Subjected to antimicrobial susceptibility testing, the isolates displayed resistance to multiple classes of antimicrobials, including IPM (resistance rate: 84%), CAZ (96%), FEP (84%), and LEV (96%) (Table 1). Among the isolates, all were identified as MDR and 21 were carbapenem-resistant. In addition, ten isolates were identified as putative hvKp based on the presence of virulence genes, and seven were cr-hvKp.

### 3.2. Phylogenetic Relationships of CC15 K. pneumoniae in Our Hospital

To assess the phylogenetic relationship of the CC15 isolates, we constructed a maximum likelihood phylogenetic tree based on 13,140 cgSNPs. We identified four lineages, two of which (Lineage 3 and Lineage 4) included only one isolate (Figure 1). The other two lineages (Lineage 1 and Lineage 2) included, respectively, seventeen and six isolates. Lineage 1 and 2 could be further divided into two sublineages each: Lineage 1a (10 isolates), 1b (7), 2a (3) and 2b (3). These results indicate the existence of clonal transmission of different lineages among different wards of our hospital.

### 3.3. Nosocomial Reservation and Transmission of CC15 Lineages

**Figure 2:**
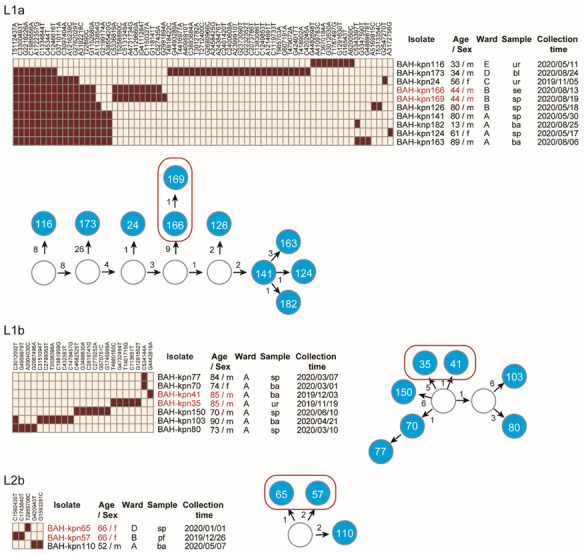
Genetic variation and nosocomial transmission of CC15 sublineages Lineage 1a, 1b, and 2b. The heatmaps represent the cgSNP loci according to the reference genome and the red blocks represent mutations from the reference. The blue circles represent the CC15 isolates and the white ones represent the potential intermediate nodes or estimated ancestors. The blue circles in a red block are from the same patients. The arrows represent the estimated relationship of parent to offspring, and the numbers represent the number of mutations. Six wards are associated (A: department of respiratory medicine; B: intensive care unit; C: department of neurological rehabilitation I; D: department of neurosurgery; E: department of neurological rehabilitation II; F: department of spinal and neural functional reconstruction).

The isolates were obtained from patients hospitalized in different wards, and the infections are likely to have been caused by different transmission events. In order to discover the relationships of the lineages we calculated the genetic differences. The number of pairwise cgSNP differences amongst the four sublineages, Lineage 1a, 1b, 2a, and 2b, were respectively 1-43, 2-13, 129-279 and 3-4. In addition, by performing a phylogenetic analysis with 665 published CC15 genomes, we found that isolates of Lineage 1a, 1b, and 2b still appear clustered (Figure S1). These results indicate clonal transmission of at least the three sublineages Lineage 1a, 1b, and 2b in our hospital (Figure 2). Based on the SNP pattern, we estimated the evolutionary history of the isolates in these sublineages. The potential intermediate nodes were estimated based on the shared SNPs.

Lineage 1a isolates spread in ten months in five wards: the department of respiratory medicine, the intensive care unit, the departments of neurological rehabilitation 1 and 2, and the department of neurosurgery. During the ten months, five of the nine patients from which the isolates were obtained were hospitalized in four departments, from which no direct epidemiological links were discovered. The seven Lineage 1b isolates were obtained from the department of respiratory medicine in eight months, and the three Lineage 2b isolates were obtained from two patients hospitalized in different wards in a time interval of four months. The cgSNP differences between the isolates and estimated intermediate nodes were mostly fewer than ten. These results indicate that there might not have been direct transmission between patients with no epidemiological links. The CC15 clones might instead spread widely and remain in the medical environment, or be carried by asymptomatic patients, and only cause infections occasionally. In addition, from three patients, we obtained two isolates from separate samples from different body systems (Figure 2). The isolates obtained from the same patients belonged to the same sublineage, and differed for 1, 3, and 6 cgSNPs from each other. These results demonstrate the intra-host variation of these sublineages and the potential intra-host transmission between different body parts.

### 3.4. Prevalence of Virulence Genes, Resistance Genes, and Plasmid Types

Resistance genes conferring resistance to at least four classes of antimicrobials were identified among all of the 25 CC15 isolates, in line with the MDR phenotype. Resistance genes including *fosA* (conferring resistance to fosfomycin), *oqxA,* and *oqxB* (conferring resistance to fluoroquinolone) were identified in all of the isolates (Figure 1). The genes encoding beta-lactamases were also prevalent, including *bla*_SHV-28_, present in 24 isolates, and *bla*_TEM-1B_, present in 21 isolates. The gene *bla*_KPC-2_ (conferring resistance to carbapenems) was harbored by eighteen isolates, including sixteen of the seventeen Lineage 1 isolates, the two Lineage 3, and Lineage 4 isolates. All of the six Lineage 2 isolates were *bla*_KPC-2_-negative and susceptible to carbapenems. Several other resistance genes were also present in specific lineages, for instance *bla*_CTX-M-15_ was mostly present in Lineage 2 and Lineage 1a, *bla*_OXA-1_ was only harbored by Lineage 1a, and *aac(6′)-IId* and *bla*_OXA-232_ were only found in Lineage 2b.

At least one of the five virulence genes *peg-344*, *iroB*, *iucA*, *rmpA*, and *rmpA2* was identified in ten isolates (Figure 1). Nine isolates, including six of the seven Lineage 1b isolates and three Lineage 2b isolates, had *iucA*, *peg-589* and *rmpA2*, while Lineage 4 isolate only harbored *iucA*. These ten isolates were therefore denoted as putative hvKp. In contrast, no virulence genes were discovered in fifteen isolates, which were denoted as cKp. Nine plasmid replicon types were identified. IncFIB replicon was present in all the 25 isolates except the one Lineage 3 isolate, IncHI1B replicon was found in Lineage 1b and Lineage 2 isolates, in line with the carriage of virulence genes. Other replicon varied in different lineages.

### 3.5. Varied Virulence Plasmid in Different CC15 Lineages

**Figure 3:**
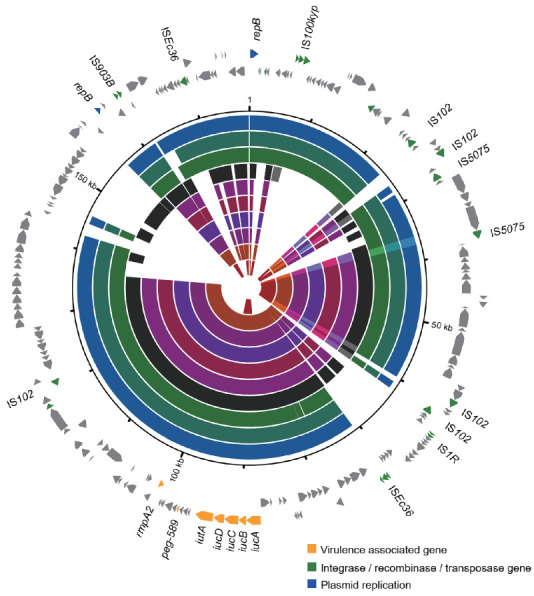
Comparison of virulence plasmid sequences found in this study with the virulence plasmid pLVPK. The outermost circle of arrows indicates the genes of reference plasmid pLVPK used for comparison (orange: virulence associated genes; green: integrase, recombinase, and transposase genes; blue: plasmid replicon genes; gray: other functions). The colored rings, from outer to inner, represent the sequences of isolates BAH-kpn65, BAH-kpn57, BAH-kpn110, BAH-kpn77, BAH-kpn103, BAH-kpn150, BAH-kpn35, BAH-kpn41, BAH-kpn70, and BAH-kpn28.

We further compared the sequences of the ten putative hvKp isolates with the classic virulence plasmid pLVPK (Figure 3). The virulence plasmid sequences of the three Lineage 2b isolates were almost the same and covered 87% of the pLVPK plasmid, having two large deletions. Among the six Lineage 1b isolates, the virulence plasmid sequences were also nearly the same and covered 68% of pLVPK, with the exception of one isolate (77% coverage). The virulence plasmid backbone of these isolates was similar to pLVPK, and contained the fragment including *rmpA2*, *peg-589*, *iutA*, and *iucABCD*. However, only *iucA* was present in the single Lineage 4 isolate, and its sequence only covered 25% of pLVPK. These results reveal lineage-specific patterns of virulence plasmid, suggesting there may be potential different sources of the virulence plasmids among different lineages.

## 4. Discussion

The emergence and prevalence of resistant *K. pneumoniae* has become one of the most urgent clinical crises. Particularly worrying is the spread of strains harboring genes encoding carbapenemases like *bla*_KPC_, which confer resistance to multiple classes of antimicrobials including cephalosporins, carbapenems, penicillins, and monobactams [22]. Since first reported, *bla*_KPC_-positive strains have spread widely, and many subtypes of the genes have been discovered. While CC258, mainly consisting of ST258, ST11, ST340, and ST512, has spread extensively throughout the world, several non-CC258 subgroups have also become high-risk clones (e.g., ST147 and ST307) and have caught the clinicians’ attention [23]. These subtypes are prevalent in healthcare settings, forming a complicated pool of intra- and inter-species gene exchange. In this study, we found that CC15 is the second prevalent subgroup in our hospital following CC258 (unpublished data), with a high rate of *bla*_KPC_ and other resistance genes. In addition, the isolates belonged to several lineages and some of them caused outbreaks in different wards, indicating that the isolates had widely spread and had remained active in our hospital.

Traditionally *K. pneumoniae* has been distinguished into two pathotypes, hvKp and cKp [24]. Between them, cKp features high antimicrobial resistance and has been prevalent in healthcare settings for decades. In contrast, hvKp has been known to cause community-associated infections with low antimicrobial resistance. However, the adaptation and prevalence of hvKp in healthcare settings has become a recent threat [5,25], worsened by the high resistance of clinical strains [24,26,27]. While hvKp subtypes like CC23 commonly causing community-acquired infections have been more and more common in hospitals in recent years [28], a high proportion of CC258 isolates have been acquiring virulence plasmid becoming hvKp [3,4]; it was also observed that other common subtypes of cKp have obtained the virulence plasmid [5], becoming high antimicrobial-resistant hvKp themselves [26]. In this study, we found that among the four CC15 lineages, two clades in two different lineages obtained the pLVPK-like virulence plasmid independently, and one of them had the *bla*_KPC-2_ gene, becoming cr-hvKp. These results demonstrate that the spreading of virulence plasmids among cKp subtypes might become easier and more common. Further experimental studies are needed to explore the mechanisms by which the plasmid is spreading, particularly among the newly emerging non-CC258 subgroups. In addition, we observed no direct epidemiological link among most of the cases, but close genetic relationships of the isolates, indicating that there were nosocomial transmissions and that the environment or other carriers might act as intermediary agents in the transmission. This is in line with recent findings that the human gut can serve as a reservoir of hvKp [29].

## 5. Conclusions

In this study, we found that multiple lineages of CC15 cr-hvKp have emerged in our hospital and have caused nosocomial transmission continually. These findings highlight the importance of surveillance for these local epidemics of non-CC258 subgroups in hospitals.

## Figures and Tables

**Table 1:** Antimicrobial susceptibility testing results.

Isolate	AMK *	CIP	IPM	LEV	TOB	AMP	SAM	ATM	FEP	CTT	CAZ	CRO	TZP	SXT
BAH-kpn163	S **	R	R	R	R	R	R	R	R	R	R	R	R	S
BAH-kpn182	S	R	R	R	R	R	R	R	R	R	R	R	R	S
BAH-kpn141	S	R	R	R	R	R	R	R	R	R	R	R	R	S
BAH-kpn124	S	R	R	R	R	R	R	R	R	R	R	R	R	S
BAH-kpn126	S	R	R	R	R	R	R	R	R	R	R	R	R	S
BAH-kpn169	S	R	R	R	I	R	R	R	R	R	R	R	R	S
BAH-kpn166	S	R	R	R	R	R	R	R	R	R	R	R	R	S
BAH-kpn24	S	R	R	R	R	R	R	R	R	R	R	R	R	S
BAH-kpn173	S	R	R	R	R	R	R	R	R	R	R	R	R	S
BAH-kpn116	S	R	R	R	R	R	R	R	R	R	R	R	R	S
BAH-kpn77	R	R	R	R	R	R	R	R	R	R	R	R	R	S
BAH-kpn70	R	R	R	R	R	R	R	R	R	I	R	R	R	S
BAH-kpn35	R	R	R	R	R	R	R	R	R	R	R	R	R	S
BAH-kpn41	R	R	R	R	R	R	R	R	R	I	R	R	R	S
BAH-kpn150	R	R	R	R	R	R	R	R	I	I	R	R	R	S
BAH-kpn80	S	R	R	R	S	R	R	R	R	R	R	R	R	R
BAH-kpn103	R	R	R	R	R	R	R	R	R	I	R	R	R	S
BAH-kpn6	S	R	S	R	I	R	R	R	S	S	R	R	S	R
BAH-kpn26	S	R	S	R	I	R	R	R	R	S	R	R	S	R
BAH-kpn12	S	R	S	R	I	R	R	S	S	S	S	R	S	R
BAH-kpn65	S	R	R	R	S	R	R	R	R	R	R	R	R	S
BAH-kpn110	R	R	R	R	R	R	R	R	R	R	R	R	R	R
BAH-kpn57	4	R	R	R	R	R	R	R	R	S	R	R	R	R
BAH-kpn144	S	R	R	R	S	R	R	R	R	I	R	R	R	S
BAH-kpn28	S	R	S	S	I	R	R	R	S	S	R	R	S	R

* Amikacin (AMK), Ciprofloxacin (CIP), Imipenem (IPM), Levofloxacin (LEV), Tobramycin (TOB), Ampicillin (AMP), Ampicillin/Sulbactam (SAM), Aztreonam (ATM), Cefepime (FEP), Cefotetan (CTT), Ceftazidime (CAZ), Ceftriaxone (CRO), Piperacillin/Tazobactam (TZP), and Trimethoprim/Sulfamethoxazole (SXT). ** Resistant I, susceptible (S), and intermediate (I).

## Data Availability

All sequencing data have been deposited in the NCBI GenBank database under accession number PRJNA881845 (https://www.ncbi.nlm.nih.gov/bioproject/?term=PRJNA881845+ accessed on 10 October 2022).

## Supplementary Materials

The following supporting information can be downloaded at: https://www.mdpi.com/article/10.3390/pathogens11101202/s1, Figure S1: Phylogenetic tree of the isolates we obtained in this study with 665 published CC15 genomes downloaded from GenBank.
