# Supplementary material for: Clonal Dissemination of Multidrug-Resistant and Hypervirulent Klebsiella pneumoniae Clonal Complex in a Chinese Hospital
Source: Pathogens. 2022 Oct 18;11(10):1202. doi: 10.3390/pathogens11101202 (PMC9607059; doi:10.3390/pathogens11101202)

Figure S1. Phylogenetic tree of the isolates we obtained in this study with 665 published CC15 genomes downloaded from GenBank. The branches of our isolates are in red.

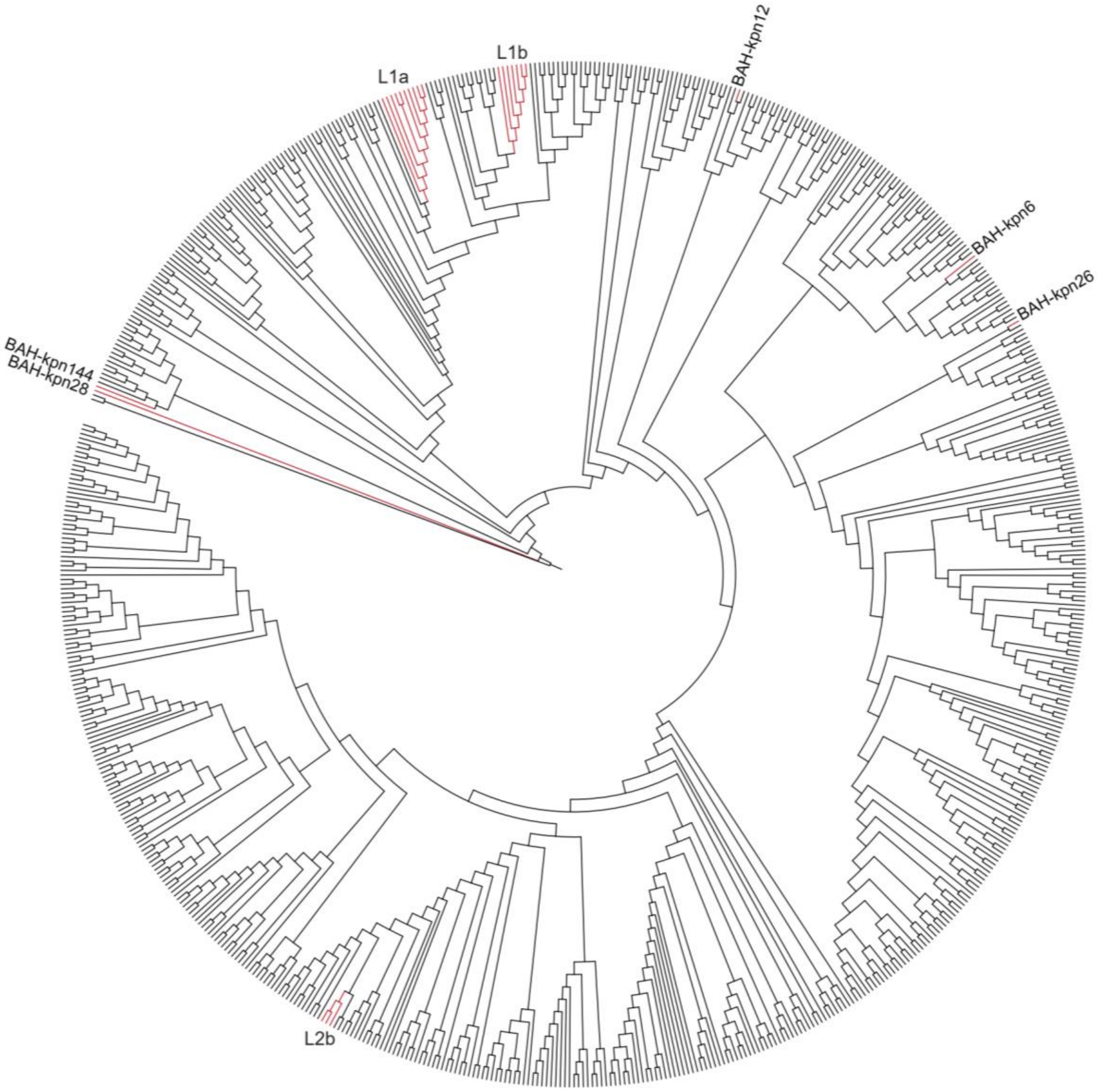

Supplement: Supplementary file 1 [file pathogens-11-01202-s001.zip › pathogens-1967739-Figure S1.pdf]
